# Nitrogen and phosphorus additions alter soil N transformations in a Metasequoia glyptostroboides plantation

**DOI:** 10.3389/fpls.2024.1448356

**Published:** 2024-08-27

**Authors:** Youzheng Zhang, Pengcheng Jiang, Yaolin Guo, Ming Wu, Xuexin Shao, Hengtao Xu, Tonggui Wu, Wenwen Yuan, Niu Li

**Affiliations:** ^1^ Key Laboratory of Engineering Oceanography, Key Laboratory of Nearshore Engineering Environment and Ecological Security of Zhejiang Province, Second Institute of Oceanography, Ministry of Natural Resources, Hangzhou, China; ^2^ Wetland Ecosystem Research Station of Hangzhou Bay, Research Institute of Subtropical Forestry, Chinese Academy of Forestry, Hangzhou, China; ^3^ School of Life Sciences, Fudan University, Shanghai, China

**Keywords:** Metasequoia glyptostroboides, nutrient enrichment, nitrification, denitrification, ammonia-oxidizing bacteria

## Abstract

**Introduction:**

Nitrogen (N) and phosphorus (P) enrichment due to anthropogenic activities can significantly affect soil N transformations in forest ecosystems. However, the effects of N and P additions on nitrification and denitrification processes in *Metasequoia glyptostroboides* plantations, and economically important forest type in China, remain poorly understood.

**Methods:**

This study investigated the responses of soil nitrification and denitrification rates, as well as the abundances of nitrifiers and denitrifiers, to different levels of N and P additions in a 6-year nutrient addition experiment in a *M. glyptostroboides* plantation.

**Results:**

Stepwise multiple regression analysis was used to identify the main predictors of nitrification and denitrification rates. The results showed that moderate N addition (N2 treatment, 2.4 mol·m^-2^) stimulated nitrification rates and abundances of ammonia-oxidizing archaea (AOA) and bacteria (AOB), while excessive N and P additions inhibited denitrification rates and reduced the abundance of *nirS*-type denitrifiers. AOB abundance was the main predictor of nitrification rates under N additions, whereas microbial biomass carbon and nirS gene abundance were the key factors controlling denitrification rates. Under P additions, tree growth parameters (diameter at breast height and crown base height) and AOB abundance were the primary predictors of nitrification and denitrification rates.

**Discussion:**

Our study reveals complex interactions among nutrient inputs, plant growth, soil properties, and microbial communities in regulating soil N transformations in plantation forests. This study also offers valuable insights for formulating effective nutrient management strategies to enhance the growth and health of *M. glyptostroboides* plantations under scenarios of increasing elevated nutrient deposition.

## 1 Introduction

Nitrogen (N) and phosphorus (P) are key limiting nutrients for productivity and health in terrestrial ecosystems. They play crucial roles in plant physiological and biochemical processes, such as photosynthesis, respiration, and protein synthesis, and influence ecosystem species composition, nutrient cycling, and diversity maintenance (Luo et al., 2022; Peñuelas and Sardans, 2022). However, owing to human activities, particularly increases in fossil fuel combustion and agricultural production, atmospheric N deposition and P pollution have increased substantially, altering the natural balance of N and P (Goll et al., 2022). Long-term excessive N and P inputs can lead to a series of ecological problems, including soil acidification, nutrient imbalance, and biodiversity loss (Jamil et al., 2022; Sun et al., 2022; Ali et al., 2023).

Soil nitrification and denitrification are key processes regulating N transformation and loss in ecosystems (Wang et al., 2022; Gineyts and Niboyet, 2023). Nitrification encompasses the aerobic transformation of ammonium into nitrite and subsequently nitrate, a sequence primarily orchestrated by chemoautotrophic bacteria and archaea harboring the *amoA* gene. This gene encodes the essential ammonia monooxygenase enzyme, initiating the pivotal first stage of nitrification (Shi et al., 2023a; Gineyts and Niboyet, 2023). Conversely, denitrification, occurring under anaerobic conditions, involves the reduction of nitrate (NO_3_^-^) into gaseous nitrogen species like nitric oxide (NO), nitrous oxide (NO_2_), and ultimately, dinitrogen gas (N_2_). Denitrifying bacteria are paramount to this process. Notably, the reduction of nitrite (NO_2_^-^) to nitric oxide (NO), facilitated by nitrite reductases encoded by the *nirK* or *nirS* genes, stands as a cornerstone in denitrification. These genes serve as reliable markers for quantifying and elucidating the abundance and community structure of denitrifiers across diverse ecosystems (Bárta et al., 2010; Kou et al., 2021). These two processes significantly affect soil N mineralization, nitrate leaching, and N_2_O emissions (Lang et al., 2021; Proctor et al., 2023). However, the effects of N and P additions on soil nitrification and denitrification are inconsistent across studies. Liu Y. et al. (2023) found that moderate N addition significantly promoted soil nitrification and denitrification in a subtropical evergreen broad-leaved forest. By contrast, Wang et al. (2021) reported that long-term high-level N fertilization, despite increasing ammonium and nitrate contents, inhibited nitrification and denitrification processes. Such discrepancies may be related to factors such as soil physicochemical properties, site conditions, and fertilization rates and duration (Baer et al., 2023; Lu et al., 2023). Furthermore, most studies focus on the effects of N addition alone, and the effects of N and P imbalance on soil N cycling remain unclear, with limited evidence, especially in plantation ecosystems.

*Metasequoia glyptostroboides* (*M. glyptostroboides*) is a rare and economically important tree species endemic to China. It is a deciduous conifer naturally distributed in the middle and lower reaches of the Yangtze River (29°10′–31°37′N, 106°50′–121°48′E) (Kato-Noguchi et al., 2023). As a living fossil with a history of over 60 million years, *M. glyptostroboides* plays essential ecological roles in maintaining regional biodiversity, regulating microclimates, conserving water and soil, and providing habitats for various organisms (Juvik et al., 2015). However, due to its limited natural distribution and historical overcutting, *M. glyptostroboides* is listed as an endangered species on the IUCN Red List. To meet the growing demand for timber and restore *M. glyptostroboides* populations, large-scale plantations have been established in China, covering an area exceeding 150,000 hectares. However, some young and middle-aged plantations are experiencing declines in productivity, which may be attributed to factors such as insufficient nutrient supply, improper management, pests and diseases, and adverse environmental conditions (Fu et al., 2023; Luo et al., 2023). Therefore, to optimize fertilization strategies and maintain plantation health and productivity, it is critical to investigate the response mechanisms of soil–plant systems to exogenous nutrient inputs in *M. glyptostroboides* plantations. Moreover, compared with natural forests, plantation ecosystems have relatively fragile structures and functions, increasing their susceptibility to environmental stressors (Bussotti and Pollastrini, 2021; Feng et al., 2022).

In this study, we conducted a 6-year nutrient addition experiment in a 6-year-old *M. glyptostroboides* plantation in eastern China. We established five N addition levels (0, 0.8, 2.4, 4.0, and 4.8 mol·m^–2^) and five P addition levels (0, 0.05, 0.2, 0.6, and 1.0 mol·m^–2^) to investigate changes in soil nitrification and denitrification rates and abundances of related functional microbes under different nutrient addition treatments. The aim of the study was to answer the following questions: (1) How do different levels of N and P additions affect soil nitrification and denitrification rates and abundances of nitrifying and denitrifying microbes in an *M. glyptostroboides* plantation? (2) What are the relations between changes in plant growth and soil chemical properties induced by N and P additions and soil N transformation processes? We hypothesized that moderate N addition would stimulate nitrification and denitrification rates by increasing soil N availability and promoting the growth of nitrifying and denitrifying microbes. However, excessive N fertilization was hypothesized to lead to soil acidification, nutrient imbalance, and toxic substance accumulation, ultimately inhibiting soil N transformation processes. P addition was also hypothesized to influence soil N transformations by regulating soil chemical properties and plant growth.

## 2 Materials and methods

### 2.1 Site description

**Figure 1:**
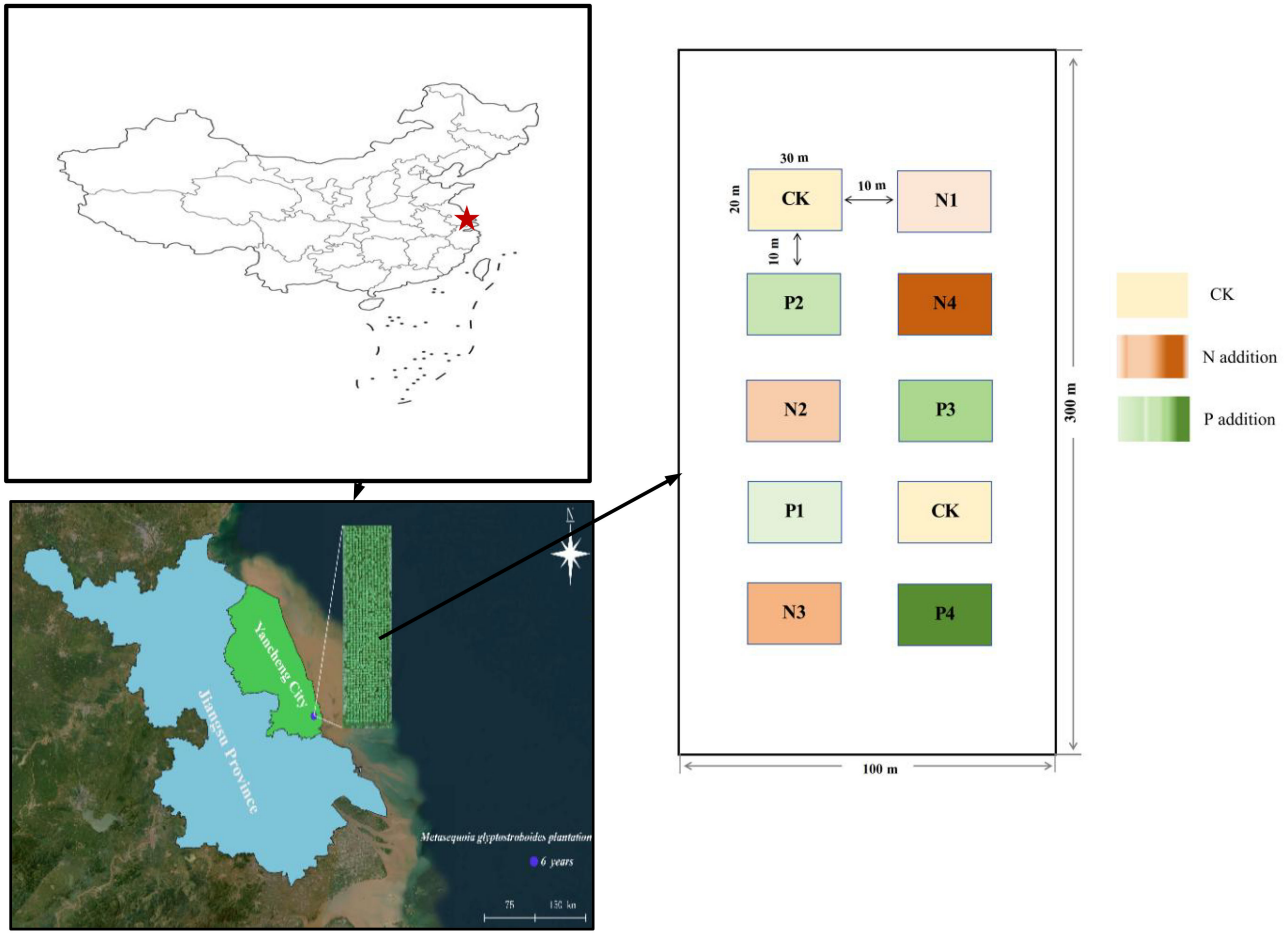
Location of Metasequoia glyptostroboides plantation in the Huanghai Forest Farm in eastern Jiangsu Province, China.

The experimental site is in the Huanghai Forest Farm (32°33′–32°57′N, 120°07′–120°53′E) in eastern Jiangsu Province, China (**Figure 1**). The regional climate is monsoon subtropical moist marine, with a mean annual temperature of 14.5°C, precipitation of 1,055.7 mm. The study site has alkaline sandy soil, which is less fertile than the yellow earth soils in the natural range of *M. glyptostroboides*. The site was the pioneer area for planting *M. glyptostroboides* in coastal China (Wang et al., 2022) and the basic situation of *M.glyptostroboides* plantations plot is shown in **Supplementary Table S1**. The study site had never been fertilized before 2015. The mean rate of N wet deposition is about 13.69 kg N ha^–1^ yr^–1^ (Yu et al., 2019) and the mean rate P wet deposition is 0.21 kg P ha^–1^ yr^–1^ (Zhu et al., 2016) in this region.

### 2.2 Experimental design

In April 2015, a completely randomized fertilization experiment with three replicate plots (300 m × 100 m) for N or P fertilizer treatments was established in a 6-year-old *M. glyptostroboides* plantation. The N fertilizer was applied as urea (CO(NH_2_)_2_ (N content 46.67%) in subplots (20 m × 30 m) with a 10-m buffer zone between adjacent plots to minimize edge effects. The selection of N and P addition levels was based on regional N and P deposition data and potential management practices in *M. glyptostroboides* plantations (Wen et al., 2022).

The five N addition treatments were the following: CK (blank control, no N addition), N1 (0.8 mol·m^–2^), N2 (2.4 mol·m^–2^), N3 (4.0 mol·m^–2^), and N4 (4.8 mol·m^–2^). Except for CK, all N addition treatments received 0.05 mol·m^–2^ P fertilizer (Ca(H_2_PO_4_)_2_) to avoid P limitation on forest growth. The P fertilizer was applied as Ca(H_2_PO_4_)_2_ (P content 26.5%) also in subplots (20 m × 30 m), with the following five P addition treatments: CK (blank control, no P addition), P1 (0.05 mol·m^–2^), P2 (0.2 mol·m^–2^), P3 (0.6 mol·m^–2^), and P4 (1.0 mol·m^–2^). Except for CK, all P addition treatments received 0.8 mol·m^–2^ N fertilizer to avoid N limitation on forest growth, which is considered sufficient for maintaining normal tree growth in this region. In each of the three main plots, there were 10 subplots, with five for N addition treatments and five for P addition treatments, for a total of 30 samples (5 treatments × 2 fertilization gradients × 3 replicate plots). Beginning in 2015, fertilizers was dissolved in 30 L of groundwater and then, applied to the corresponding plots near the soil surface using a backpack sprayer during the growing period for each treatment: 60% at the beginning of April and 40% in mid-June.

### 2.3 Sampling collection and analysis

Soil samples were collected in October 2021, six years after the initial fertilization experiments. This timing was selected to provide ample duration to assess the long-term impacts of fertilization on N cycles and microbial communities. Additionally, mid-autumn presents a critical juncture characterized by slow plant growth rates coupled with heightened microbial metabolic activity. In each plot, five soil cores (0–10-cm depth) were randomly collected using a 5-cm diameter soil auger and mixed to form a composite sample. Collected samples were stored in an icebox and transported to the laboratory. Samples were sieved (< 2 mm) and divided into three subsamples. One subsample was stored at 4°C for analyses of potential nitrification and denitrification rates. One subsample was air-dried and subsequently analyzed for soil physical-chemical properties. The remaining subsample was immediately stored at −80°C for DNA analysis.

Soil moisture was determined as the mass lost after heating at 70°C for 48 h. Soil pH was measured using a pH meter (PHSJ-3F, Shanghai INESA Scientific Instrument Co., Ltd., Shanghai, China) with soil-to-water ratio of 1:2.5 (Takamoto et al., 2023). Soil salinity was determined by measuring the electrical conductivity of a soil-water extract (1:5 ratio) using a conductivity meter (DDS-307, Shanghai INESA Scientific Instrument Co., Ltd., Shanghai, China) (Smith and Doran, 1996). Nitrate (NO_3_^–^) and ammonium (NH_4_^+^) were extracted with a 2.0 M KCl solution and measured using a continuous flow analyzer (SAN^++^, Skalar Analytical B.V., Breda, the Netherlands). Total nitrogen (TN) was determined using a CN analyzer (Vario Max, Elementar Analysensysteme GmbH, Langenselbold, Germany) (Yao et al., 2019). Total phosphorus (TP) was determined using a phosphomolybdate blue method (Xie et al., 2013). Soil organic carbon (SOC) was determined using a potassium dichromate oxidation-colorimetric technique (Liu X. et al., 2023). Microbial biomass carbon (MBC) and nitrogen (MBN) were determined using the chloroform fumigation-extraction method (Vance et al., 1987).

Tree growth was evaluated by measuring the diameter at breast height (DBH) of all trees in each plot (Li et al., 2015). Crown base height (CBH) was determined by measuring the distance from the ground to the lowest live-branch whorl using a laser rangefinder. Tree height was measured using a clinometer (Kim et al., 2009).

### 2.4 Determinations of soil nitrogen transformation rates

Potential nitrification rates were determined using a shaken soil-slurry method, as described by Guo et al. (2017) and Li et al. (2020b) with minor modifications. In brief, fresh soil samples, 15 g, were transferred to sterile 250-mL Erlenmeyer flasks, followed by the addition of 100 mL of 1.0 mM phosphate buffer (pH 7.2) supplemented with 1.0 mM NH_4_^+^. Flasks were incubated on an orbital shaker at 200 rpm and 25°C for 24 h in the dark. During the incubation period, 10-mL aliquots of soil slurry were collected at 2, 4, 12, 22, and 24 h. Aliquots were centrifuged at 8,000 × *g* for 5 min, and the supernatants were filtered for determination of nitrite (NO_2_^–^) and nitrate (NO_3_^–^) concentrations. Potential nitrification rate was estimated by performing a linear regression analysis of the combined NO_2_^–^ and NO_3_^–^ concentrations as a function of time.

To determine potential denitrification rates, soil slurry experiments were conducted (Xue et al., 2020). The collected sediment samples were combined with deionized water in a 1:7 ratio and purged with helium for 30 min, with stirring to create homogenized slurries. The resulting slurries were transferred into 12-mL glass vials (Exetainer, Labco, High Wycombe, UK) and sealed with butyl rubber stoppers. The vials were then subjected to a 36-h pre-incubation period in the dark at the corresponding *in situ* temperature (25°C in October) to deplete any residual NO_2_^–^, NO_3_^–^, and oxygen (Hou et al., 2015). After the pre-incubation, slurry vials were amended with 100 μL of helium-purged ^15^NO_3_^–^ stock solutions (99% ^15^N, Cambridge Isotope Laboratories, Inc., Andover, MA, USA) to achieve a final ^15^N concentration of 100 μmol L^–1^ in each vial. The incubated soil slurries were divided into two groups: initial samples and final samples. The initial samples were immediately terminated by adding 200 μL of 50% ZnCl_2_ solution, while the final samples were incubated for an additional 8 h before being fixed with the same ZnCl_2_ solution. The concentrations of nitrogen gases (^29^N_2_ and ^30^N_2_) in each vial were quantified using membrane inlet mass spectrometry (MIMS) (GAM200, IPI, Bremen, Germany). Potential denitrification rates were calculated based on the equation proposed by Thamdrup and Dalsgaard (2002).

### 2.5 DNA isolation and quantitative PCR

Soil DNA was extracted from frozen samples using Powersoil™ DNA Isolation Kits (MOBIO Laboratories, Inc., Carlsbad, CA, USA) following the manufacturer’s protocol. The abundances of nitrifier genes (ammonia-oxidizing archaea (AOA) *amoA* and ammonia-oxidizing bacteria (AOB) *amoA*) and denitrifier genes (*nirK* and *nirS*) were quantified using real-time quantitative PCR (qPCR) on an ABI 7500 Detection System (Applied Biosystems, Foster City, CA, USA) with the SYBR Green approach. The primer sets employed were Arch-amoAF (5’-STAATGGTCTGGCTTAGACG-3’) and Arch-amoAR (5’-GCGGCCATCCATCTGTATGT-3’) for AOA *amoA* (Gao et al., 2018); amoA-1F (5’-GGGGTTTCTACTGGTGGT-3’) and amoA-2R (5’-CCCCTCKGSAAAGCCTTCTTC-3’) for AOB *amoA* (Rotthauwe et al., 1997); F1aCu (5’-ATCATGGTSCTGCCGCG-3’) and R3Cu (5’-GCCTCGATCAGRTTGTGGTT-3’) for *nirK* (Hallin and Lindgren, 1999); and cd3aF (5’-GTSAACGTSAAGGARACSGG-3’) and R3cd (5’-GASTTCGGRTGSGTCTTGA-3’) for *nirS* (Throbäck et al., 2004). The thermal profiles for each gene were as follow: AOA *amoA* and AOB *amoA*: 50°C for 2 min, 95°C for 10 min; 30 cycles of 94°C for 30 s, 56°C (AOA amoA) or 55°C (AOB amoA) for 45 s; an extension at 72°C for 5 min; *nirS*: 50°C for 2 min, 95°C for 10 min; 45 cycles of 95°C for 30 s, 58°C for 40 s; an extension at 72°C for 1 min; *nirK*: 50°C for 2 min, 10 min at 95°C; 40 cycles of 95°C for 30 s, 58°C for 40 s; an extension at 72°C for 40 s. Negative controls without DNA templates were included in each amplification run to check for contamination. Standard melting curves were generated using 10-fold serial dilutions of a plasmid containing the AOA, AOB, *nirK* or *nirS* target gene inserts. Each sample was analyzed in triplicate qPCR reactions to ensure accurate amplification, and the amplification efficiencies were 95.2% for AOA *amoA* (R^2^ = 0.998), 96.4% for AOB *amoA* (R^2^ = 0.997), 94.7% for *nirK* (R^2^ = 0.996) and 95.8% for *nirS* (R^2^ = 0.999). Gene copy numbers were calculated based on the standard curves and normalized to copies per gram of dry soil.

### 2.6 Statistical analyses

To assess the effects of N and P addition treatments on soil characteristics, tree growth indicators, nitrification and denitrification rates, and nitrifier and denitrifier gene abundances, we used a comprehensive statistical approach. For each measured parameter, mean values and standard deviations were computed based on three replicates (*n* = 3). Prior to analysis of variance (ANOVA), data were tested for normality using the Shapiro-Wilk test and homogeneity of variance using Levene’s test. If the assumptions of normality and homogeneity of variance were not met, data were log-transformed to satisfy these assumptions. The effects of N and P additions were evaluated using a one-way ANOVA, followed by Tukey’s *post hoc* tests for pairwise comparisons when significant treatment effects were detected. The significance threshold was set at *P* < 0.05 for all statistical analyses, which were conducted using the SPSS 22.0 software package (IBM Corporation, Armonk, NY, USA). To investigate the relations between nitrification and denitrification rates and various environmental factors and abundances of the functional genes of nitrifiers and denitrifiers. Pearson correlation analysis was performed using the ggplot2 package in R (v2023.06.2) (R Core Team, 2023; https://www.R-project.org/). To determine the most suitable models for explaining nitrification and denitrification rates, we used a stepwise multiple regression approach using R software. In the analysis, soil chemical properties and microbiological parameters were the predictor variables and nitrification or denitrification rate was the dependent variable. The Akaike Information Criterion (AIC), which considers both models fit and complexity by penalizing models with a higher number of parameters, was used to select the best-fitting models (Liao et al., 2018).

## 3 Results

### 3.1 Effects of nitrogen and phosphorus additions on tree growth and soil properties

**Figure 2:**
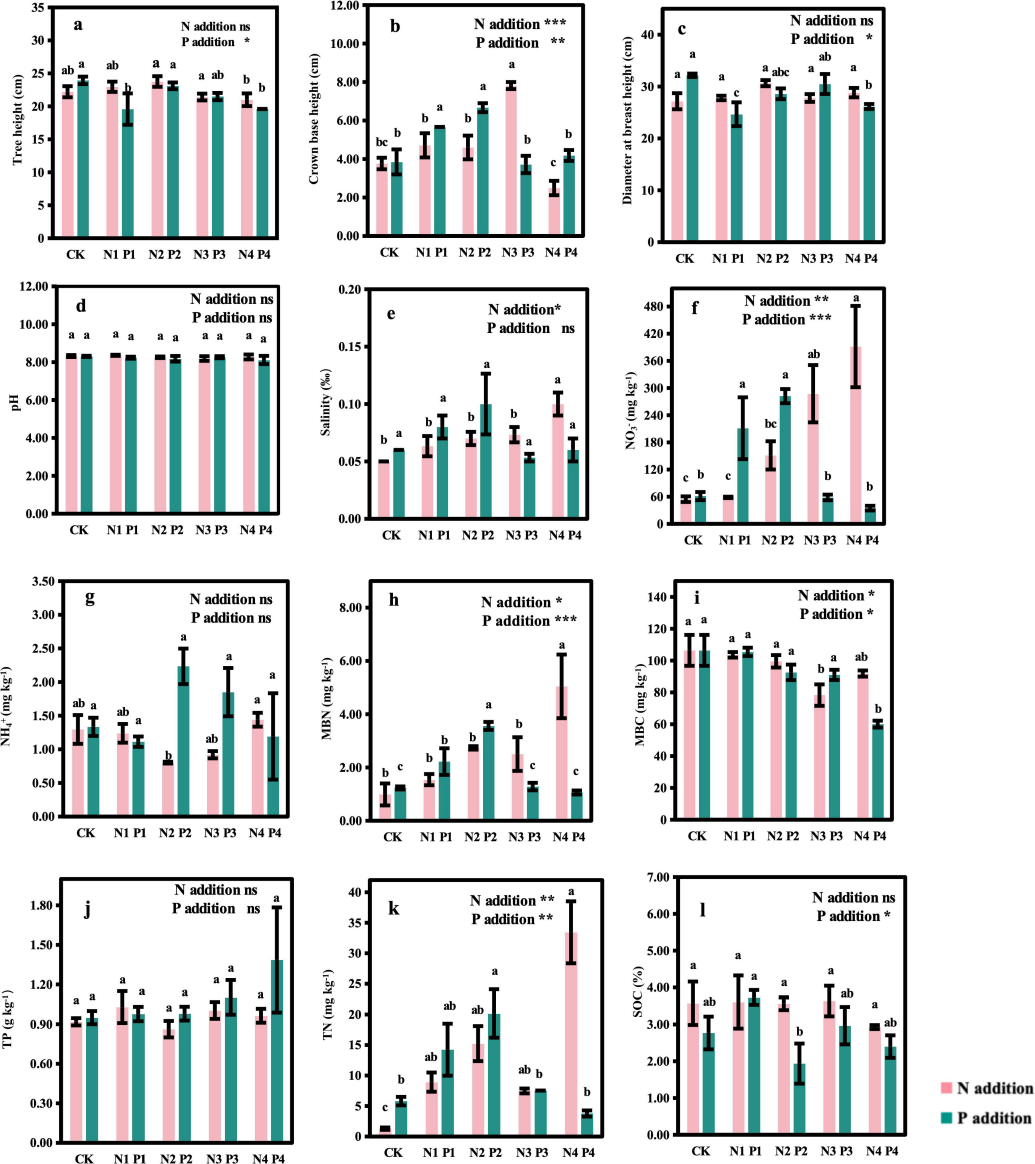
Effects of nitrogen and phosphorus additions on tree growth and soil chemical and microbial properties in a *Metasequoia glyptostroboides* plantation. CK represents the control treatment without N or P addition; N1, N2, N3, and N4 represent treatments with nitrogen additions of 0.8 mol·m^–2^, 2.4 mol·m^–2^, 4.0 mol·m^–2^, and 4.8 mol·m^–2^, respectively; P1, P2, P3, and P4 represent treatments with phosphorus additions of 0.05 mol·m^–2^, 0.2 mol·m^–2^, 0.6 mol·m^–2^, and 1.0 mol·m^–2^, respectively; Values are presented as mean ± standard deviation (SD), n = 3. Different letters indicate significant differences (P < 0.05) in plant and soil properties between different treatments. NO_3_^−^: nitrate nitrogen; NH_4_^+^: ammonia nitrogen; MBC: microbial biomass carbon; MBN: microbial biomass nitrogen; TN: total nitrogen; TP: total phosphorus; SOC: soil organic carbon. ns, *, **, and *** in each panel represent non-significant, significant at P < 0.05, P < 0.01, and P < 0.001, respectively, for the effects of N and P additions on the variables. (**A**: Tree height; **B**: Crown base height; **C**: Diameter at breast height; **D**: pH; **E**: Salinity; **F**: NO_3_^−^; **G**: NH_4_^+^; **H**: MBN; **I**: MBC; **J**: TP; **K**: TN; **L**: SOC).

N and P additions significantly affected the growth of *M. glyptostroboides* (**Figures 2A-C**). Compared to CK, N1 to N3 treatments increased CBH by 0.25, 0.22, and 1.08 times, respectively, while the CBH in the N4 treatment decreased but not significantly compared to CK (**Figure 2B**). N additions had no effect on tree height (**Figure 2A**) or DBH (**Figure 2C**). However, P additions significantly affected tree height (*P* < 0.05), CBH (*P* < 0.01), and DBH (*P* < 0.05). Compared to CK, P1 and P4 treatments significantly reduced tree height by 18% and 19% (**Figure 2A**), respectively, and reduced DBH by 23% and 19% (**Figure 2C**), respectively. In contrast, P1 and P2 treatments significantly increased CBH by 47% and 73% (**Figure 2B**), respectively, while P3 and P4 treatments had no significant effect on CBH.

N and P additions significantly influenced various soil chemical properties (**Figures 2D-L**). N additions had significant effects on soil salinity (P < 0.05), NO_3_^-^ (P < 0.01), MBN (*P* < 0.05), MBC (*P* < 0.05), and TN (*P* < 0.01). Compared to CK, soil NO_3_^-^ concentrations (**Figure 2F**) increased significantly in all N addition treatments, with the highest values observed in the N4, which were 7 times higher than CK. Similarly, soil NH_4_^+^ concentrations (**Figure 2G**) significantly increased in the N4 treatment, doubling the values of CK. Soil salinity (**Figure 2E**), MBN (**Figure 2H**), and TN (**Figure 2K**) also increased significantly in all N addition treatments compared to CK. However, there were no significant differences in pH (**Figure 2D**), NH_4_^+^ (**Figure 2G**), TP (**Figure 2J**) and SOC (**Figure 2L**) among N addition treatments. P additions significantly affected soil NO_3_^-^ (*P* < 0.001), MBN (*P* < 0.001), MBC (*P* < 0.05), TN (*P* < 0.01), and SOC (*P* < 0.05). Compared to CK, soil NO_3_^-^ concentrations (**Figure 2F**) increased by 2.5 and 3.6 times under P1 and P2 treatments, respectively, but decreased by 5% and 43% under higher P addition levels (P3 and P4). MBN content (**Figure 2H**) increased significantly by 0.8 and 1.9 times under P1 and P2 treatments compared to CK, but declined with further P additions, reaching the lowest value in P4. The lowest MBC content (**Figure 2I**) was also observed in the P4 treatment. TN content (**Figure 2K**) increased by 1.5 and 2.5 times under P1 and P2 treatments, respectively, compared to CK, but decreased with higher P levels, with the lowest TN content in P4. SOC content (**Figure 2L**) increased by 34% under P1 compared to CK, but decreased with further P additions (P2, P3, and P4). There were no significant differences in pH (**Figure 2D**), salinity (**Figure 2E**), NH_4_^+^ (**Figure 2G**) and TP (**Figure 2J**) among P addition treatments.

### 3.2 Effects of nitrogen and phosphorus additions on nitrification and denitrification

N addition significantly affected nitrification (*P* < 0.001) and denitrification rates (*P* < 0.001). The lowest nitrification rate was in N1, but the rate was not significantly different from that in CK or N3. The highest rate was observed in the N2, which was not significantly different from N4 and the both treatments showed rates approximately 2.3 and 2.2 times higher than CK, respectively. Denitrification rates generally decreased with increasing N additions, with the lowest rates observed in the N4.

**Figure 3:**
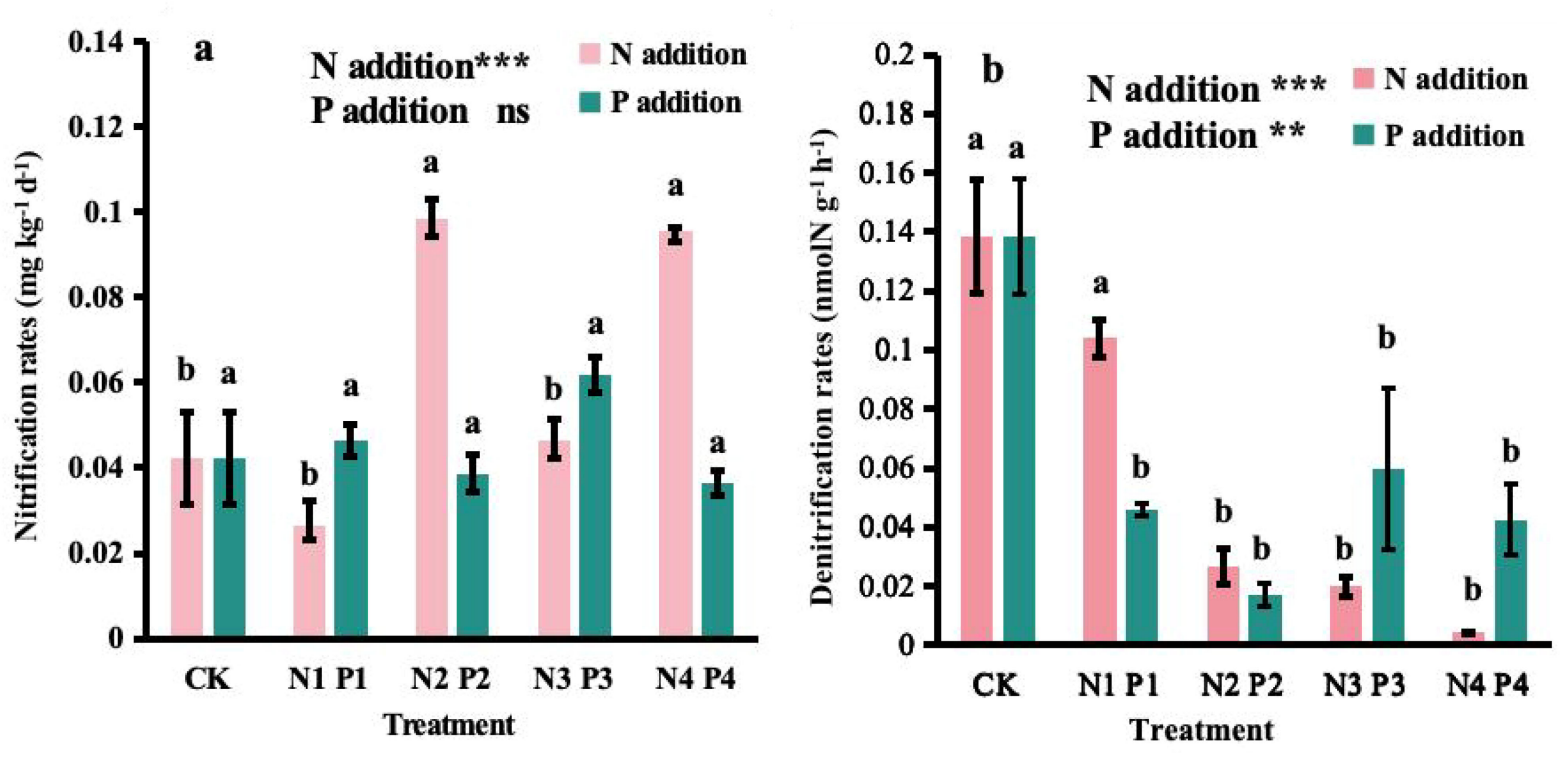
Effects of nitrogen (N) and phosphorus (P) additions on potential **(A)** nitrification rates and **(B)** denitrification rates in a Metasequoia glyptostroboides plantation. CK represents the control treatment without N or P addition; N1, N2, N3, and N4 represent treatments with nitrogen additions of 0.8 mol·m^–2^, 2.4 mol·m^–2^, 4.0 mol·m^–2^, and 4.8 mol·m^–2^, respectively; P1, P2, P3, and P4 represent treatments with phosphorus additions of 0.05 mol·m^–2^, 0.2 mol·m^–2^, 0.6 mol·m^–2^, and 1.0 mol·m^–2^, respectively. Values are presented as mean ± standard deviation (SD), n = 3. Different letters indicate significant differences (P < 0.05) in potential rates of nitrification or denitrification between different treatments. ns, **, and *** in each panel represent non-significant, significant at P < 0.01, and P < 0.001, respectively, for the effects of N and P additions on the corresponding variables.

P addition had no significant effect on nitrification rates (*P* > 0.05) but affected denitrification rates (*P* < 0.01). Compared to CK, the nitrification rates under different levels of P addition did not show significant differences (**Figure 3A**). However, the denitrification rates strikingly decreased with increasing P addition levels, with the lowest rate observed in the P2, which was 87% lower than CK (**Figure 3B**).

### 3.3 Effects of nitrogen and phosphorus additions on the abundances of nitrifiers and denitrifiers

**Figure 4:**
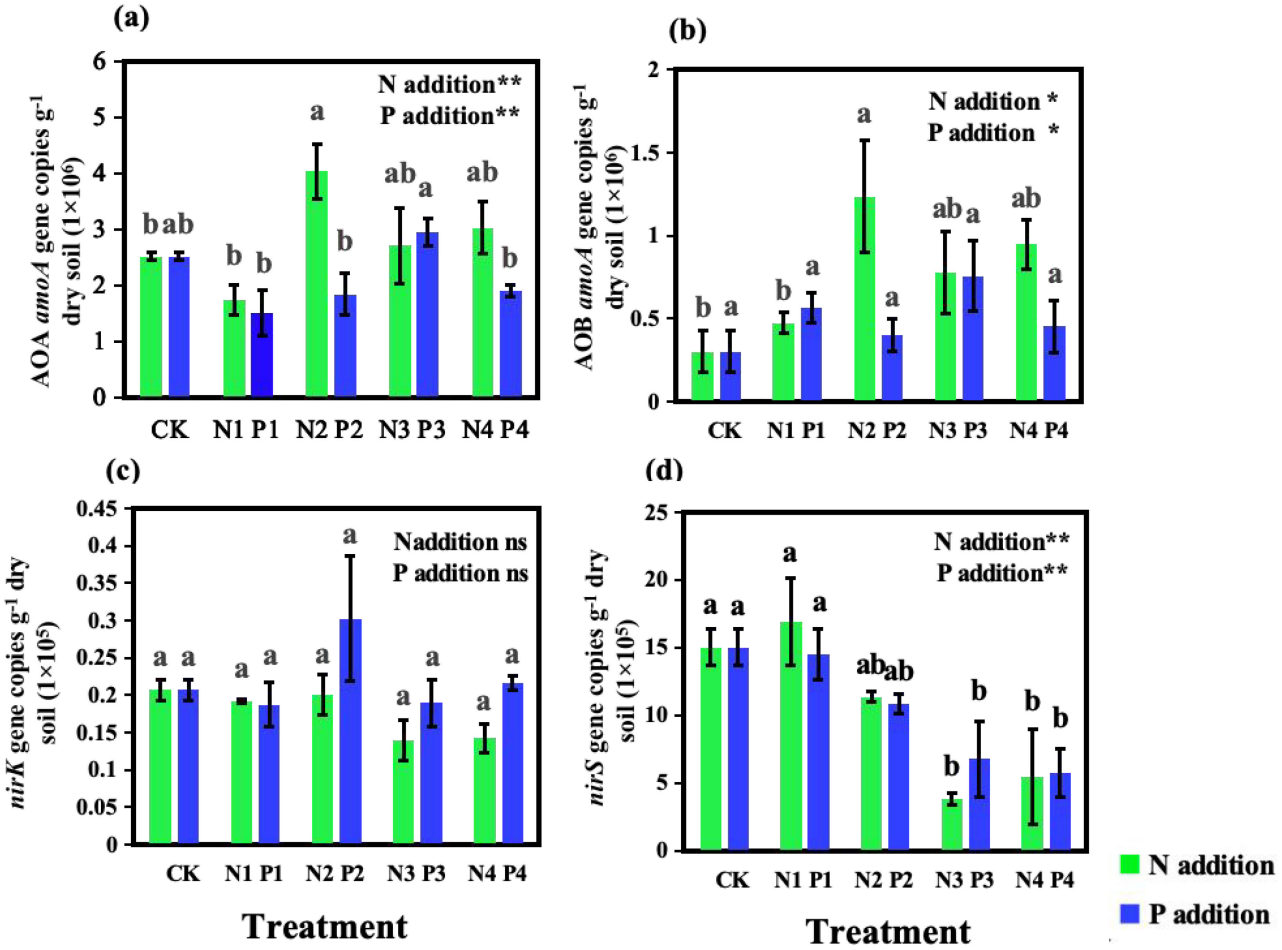
Effects of nitrogen (N) and phosphorus (P) additions on the abundances of *amoA* sequences from **(A)** ammonia-oxidizing archaea (AOA) and **(B)** ammonia-oxidizing bacteria (AOB) and denitrifier sequences **(C)** *nirK* and **(D)** *nirS* in soil under a *Metasequoia glyptostroboides* plantation. CK represents the control treatment without N or P addition; N1, N2, N3, and N4 represent treatments with nitrogen additions of 0.8 mol·m^–2^, 2.4 mol·m^–2^, 4.0 mol·m^–2^, and 4.8 mol·m^–2^, respectively; P1, P2, P3, and P4 represent treatments with phosphorus additions of 0.05 mol·m^–2^, 0.2 mol·m^–2^, 0.6 mol·m^–2^, and 1.0 mol·m^–2^, respectively. Values are presented as mean ± standard deviation (SD), n = 3. Different letters indicate significant differences (P < 0.05) in gene abundances between different treatments. ns, **, and * in each panel represent non-significant, significant at P < 0.01, and P < 0.05, respectively, for the effects of N and P additions on the corresponding variables.

N addition significantly impacted the abundance of nitrifiers. Compared to CK, N additions enhanced the abundance of both AOA *amoA* and AOB *amoA* genes (**Figures 4A, B**), with the N2 showing the highest abundances, being 1.6 times and 4 times higher than CK, respectively. In terms of denitrifier abundance, the *nirK* gene abundance remained relatively stable across all N addition treatments, showing no significant differences compared to CK (*P* > 0.05). However, *nirS* gene abundance was significantly affected by N additions (*P* < 0.01), generally decreasing with higher levels of N. The lowest *nirS* gene abundance was observed in the N3 treatment, which was a 75% reduction compared to CK.

P addition had a significant effect on AOA *amoA* abundances (*P* < 0.01) (**Figure 4A**) but did not affect AOB *amoA* gene abundances (*P* > 0.05) (**Figure 4B**). Compared to CK, the P1, P2, and P4 treatments resulted in reduction in AOA *amoA* gene abundance by 40%, 27%, and 24%, respectively. In contrast, varying levels of P additions did not significantly alter AOB *amoA* gene abundances when compared to CK (**Figure 4B**). Similar to the N treatments, P additions did not significantly impact *nirK* gene abundances but had a pronounced effect on *nirS* gene abundances. With increasing levels of P addition, *nirS* gene abundances decreased, with the P2, P3, and P4 treatments showing reductions of 28%, 55%, and 62%, respectively, compared to CK.

### 3.4 Key factors controlling nitrification and denitrification

**Figure 5:**
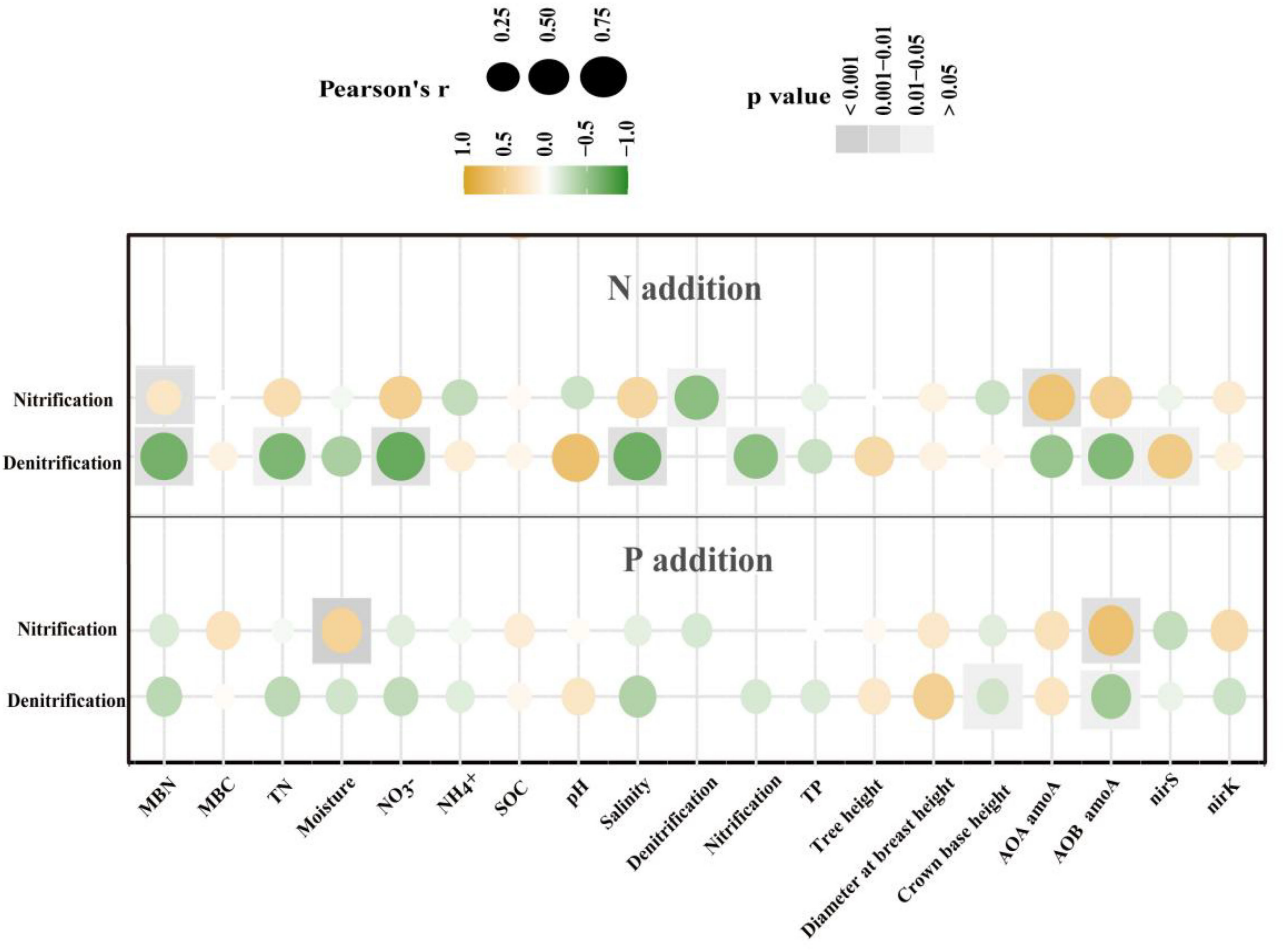
Pearson correlation analyses between potential rates of nitrification and denitrification and soil and microbial properties and indicators of tree growth under nitrogen (N) and phosphorus (P) additions in a Metasequoia glyptostroboides plantation. Correlations were evaluated with r, the correlation coefficient, ranging from –1 to +1.

Pearson correlation analysis revealed that with N additions, the main factors influencing nitrification rates were MBN and AOA *amoA* gene abundance, whereas the principal factors affecting denitrification rates were MBN, TN, NO_3_^–^, pH, salinity, and AOB *amoA* and *nirS* gene abundances (**Figure 5**). By contrast, with P additions, soil moisture and AOB *amoA* gene abundance were the primary factors controlling nitrification rates, whereas CBH and AOB *amoA* gene abundance were the key determinants of denitrification rates (**Figure 5**).

To further elucidate the complex interplay of various environmental factors and microbial communities in regulating nitrification and denitrification processes, we used stepwise multiple linear regression analysis to identify which linear combination of independent variables best predicted rates of nitrification and denitrification (**Table 1**). With N additions, AOB *amoA* gene abundance was the most significant predictor of nitrification rates, whereas for denitrification rates, MBC and abundance of the *nirS* gene were the primary factors explaining the variation. With P additions, DBH and AOB *amoA* gene abundance were the main factors predicting nitrification rates, whereas AOB *amoA* gene abundance and CBH were the key predictors of denitrification rates.

**Table 1:** Best-fit multiple linear regression models to predict nitrification and denitrification rates using soil and microbial properties and indicators of tree growth under nitrogen (N) and phosphorus (P) additions in a Metasequoia glyptostroboides plantation, with coefficient of determination (R^2^) and significance (P) values.

Treatment	Equation	R^2^	P
N addition	Nitrification = 0.024 + 0.016AOB amoA	0.452	<0.01
	Denitrification = 0.430 + 0.006MBC + 0.894 nirS	0.802	<0.001
P addition	Nitrification = –0.017 + 0.034DBH + 0.02AOB amoA	0.649	<0.01
	Denitrification = –0.809 – 0.31AOB amoA + 0.213CBH	0.451	<0.001

DBH, diameter at breast height; CBH, crown base height; MBC, microbial biomass carbon.

## 4 Discussion

### 4.1 Effects of nitrogen and phosphorus additions on tree growth and soil properties

Our results revealed that N addition did not significantly impact tree height, DBH, and CBH, except for the N3 treatment, which significantly increased CBH compared to CK. In contrast, P addition significantly influenced all these tree growth indicators (**Figures 2A-C**). This suggests that the growth indicators of *M. glyptostroboides* plantation are more sensitive to P addition than N addition. These findings align with the recent study by Mo et al. (2021), which demonstrated differential responses of forest leaf nutrient elements to N and P additions. This indicates that coastal *M. glyptostroboides* plantation exhibit distinct adaptive responses in their primary above-ground growth attributing in response to N and P fertilization.

Furthermore, our study demonstrates that N and P additions strikingly affect soil chemical and microbial properties. N additions significantly increased MBN and TN content (**Figures 2H, K**), aligning with the findings of Xiao et al. (2015) and Peng et al. (2017). The increase in soil N availability due to N additions stimulates microbial growth and activity, ultimately leading to an increase in MBN and accelerated soil N cycling (He et al., 2023). However, SOC did not respond significantly to N additions (**Figure 2L**), indicating that limited effects of N inputs on carbon sequestration in the sampled soils. This limited response may be attributed to the soils having reached a state of nitrogen saturation. In such conditions, microbial communities and vegetation are already adapted to existing nitrogen levels, and thus, additional nitrogen inputs do not further enhance carbon sequestration (Nadelhoffer et al., 1999). Moreover, the relatively short duration of our study may not have been sufficient to observe the long-term effects of nitrogen addition on SOC, which could take longer periods to manifest. In contrast, P additions had positive effects on MBN and SOC at low P levels (P1 and P2) but negative effects at higher P levels (P3 and P4) (**Figures 2H, I**), suggesting that the impact of P fertilization on soil C and N dynamics depends on the application rate (Ali et al., 2023). Low levels of P addition likely promote microbial activity and carbon accumulation, as supported by studies showing enhanced microbial activity and organic matter turnover with optimal P availability (Turner et al., 2013; Li et al., 2020a), higher levels of P may cause nutrient imbalances, potentially inhibiting microbial activity and carbon accumulation (Qin et al., 2024). Overall, our study highlights that coastal plantation forests exhibit distinct adaptive responses in the key soil chemical and microbial properties to N and P additions. Particularly, the effects of P additions are dose-dependent, underscoring the importance of considering application rates in fertilization strategies. These findings are crucial for developing effective fertilization practices, and future research should explore the long-term impacts of N and P additions to better understand their potential effects on forest ecosystems.

### 4.2 Effects of nitrogen and phosphorus additions on nitrification and denitrification

Our research underscores the nuanced impact of N and P amendments on the dynamics of nitrification and denitrification within an *M. glyptostroboides* plantation. Notably, N addition significantly bolstered nitrification rates, whereas P addition failed to elicit a marked effect, as evident in **Figure 3A**. This observation aligns with prior investigations, such as Zhang et al. (2015), who documented a substantial enhancement of soil nitrification in acidic forest soils upon N addition. Conversely, Xiao et al. (2023) concurred that P addition did not significantly alter nitrification rates. Regarding denitrification, we observed a decline in rates across all treatments except for the N1 and P1 levels (**Figure 3B**). This pattern is corroborated by Chen et al. (2023), who suggested that high N levels could reduce denitrification rates by altering soil moisture and pH. However, laboratory-based studies by Wang et al. (2018) indicated a potential stimulatory effect of P on denitrification. Thus, further field studies are essential to fully assess the effects of P on denitrification. Furthermore, our findings indicate that moderate N (N2) and P (P2) additions can substantially increase nitrification rates while decreasing denitrification rates, which may help facilitate faster nitrogen cycling in forest ecosystems and enhance the storage of plant-available nitrogen in the soil.

### 4.3 Effects of nitrogen and phosphorus additions on the abundances of nitrifiers and denitrifiers

Moderate to high levels of N addition (N2, N3, and N4) stimulated the abundances of both AOA and AOB *amoA* genes (**Figures 4A, B**), suggesting that N fertilization can promote the growth and activity of ammonia-oxidizing microorganisms in forest soils. The stimulation of nitrifier growth by N additions can be attributed to increases in substrate availability and alleviation of N limitation for microbial growth (Shi et al., 2023b).

The abundance of denitrifiers, as indicated by *nirK* and *nirS* gene abundances, showed different responses to N and P additions. Abundance of the *nirK* gene remained relatively stable across treatments, whereas that of the *nirS* gene generally decreased with increasing additions of N and P (**Figures 4C, D**). This result suggests that denitrifying microorganisms harboring *nirS* genes are more sensitive to nutrient additions than those harboring *nirK* genes. The differential responses of *nirK* and *nirS* communities to N and P additions may be due to differences in their physiological and ecological characteristics, such as their adaptability to soil environmental conditions (e.g., pH and NO_3_^-^) (Chen et al., 2022).

### 4.4 Key factors controlling nitrification and denitrification

Stepwise multiple linear regression analysis revealed that with N additions, AOB *amoA* gene abundance was the most significant predictor of nitrification rates, whereas MBC and *nirS* gene abundance were the primary factors explaining the variation in denitrification rates (**Table 1**). This result suggests that the abundance and activity of AOB *amoA* genes are critical in regulating nitrification processes in forest soils under N enrichment. Previous studies also report strong correlations between AOB *amoA* abundance and nitrification rates in N-rich soils (Song and Niu, 2022). The importance of MBC and *nirS*-type denitrifiers in controlling denitrification rates with N additions can be attributed to the close coupling between carbon availability, denitrifier community composition, and denitrification activity in forest soils (Sun and Jiang, 2022; Yang et al., 2022).

With P additions, DBH and AOB *amoA* gene abundance were the main factors predicting nitrification rates, whereas CBH and AOB *amoA* gene abundance were the key predictors of denitrification rates (**Table 1**). This result suggests that tree growth and ammonia-oxidizing bacterial communities are important drivers of nitrification and denitrification processes in forest soils under P fertilization. Previous studies report that tree growth and productivity can influence soil N cycling processes by altering soil physicochemical properties, microbial community composition, and root–microbe interactions (Rodionov et al., 2020; Wan et al., 2021). The importance of AOB in regulating both nitrification and denitrification rates with P additions highlights the central role of ammonia oxidation in controlling soil N transformations in P-rich soils (Li et al., 2023; Zhang et al., 2023).

It is important to note that, although our results highlight the crucial role of functional microbes in nitrification and denitrification processes, the findings are based on data collected from a single date within one year. Given that nitrification and denitrification rates are episodic and highly influenced by varying environmental conditions (Wu et al., 2021; Landi and Lu, 2022), relying on data from a single date may not sufficiently capture the complexities of the system. Therefore, more comprehensive temporal sampling is necessary to provide a more robust understanding of how N and P additions influence soil microbial dynamics and associated biogeochemical processes over time.

## 5 Conclusions

Our study demonstrates that N and P additions have significant and contrasting effects on plant growth, soil properties, and key N cycling processes in an *M. glyptostroboides* plantation. Moderate levels of N and P additions can enhance the growth of *M. glyptostroboides* and stimulate soil nitrification and denitrification processes. Conversely, excessive nutrient inputs can have adversely affect these systems, highlighting the importance of precisely tailored fertilization strategies to uphold the productivity and ecological sustainability of M. glyptostroboides plantations. Under N additions, the AOB abundance primarily influences nitrification rates, while microbial biomass carbon and the *nirS* gene abundance mainly control denitrification rates. Similarly, tree growth parameters and AOB abundance are the main predictors of nitrification and denitrification rates under P additions. However, further research is needed to develop specific fertilization recommendations that account for the long-term effects of nutrient additions and the potential interactions between N and P inputs.

## Data Availability

The raw data supporting the conclusions of this article will be made available by the authors, without undue reservation.
