# Supplementary material for: Nitrogen and phosphorus additions alter soil N transformations in a Metasequoia glyptostroboides plantation
Source: Front Plant Sci. 2024 Aug 27;15:1448356. doi: 10.3389/fpls.2024.1448356 (PMC11384580; doi:10.3389/fpls.2024.1448356)
Supplement: Supplementary file 1 [file Table1.docx]

**Table S1**. The basic situation of *M.glyptostroboides* plantations plot (in 2014)

| Soil physical and chemical properties | | | | | Stand structure | | |
| --- | --- | --- | --- | --- | --- | --- | --- |
| Organic carbon (g·kg^-1^) | Total nitrogen (g·kg^-1^) | Alkali-hydrolyzable nitrogen  (mg·kg^-1^) | Total phosphorus (g·kg^-1^) | Available phosphorus  (mg·kg^-1^) | Average tree height  (m) | Stand density (stems·hm^-2^) | Crown width  (m) |
| 8.19±0.38 | 0.71±0.01 | 54±4.27 | 0.85±0.03 | 14.27±3.91 | 10.33±1.83 | 417 | 4.61±0.65 |
